# PROTOCOL: The effects of flipped classrooms to improve learning outcomes in undergraduate health professional education: A systematic review

**DOI:** 10.1002/cl2.1041

**Published:** 2019-09-05

**Authors:** Cho Naing, Dinesh Kumar Chellappan, Wong Shew Fung, Amy Riegelman, Maxine A Whittaker

**Affiliations:** ^1^ International Medical University (IMU) Kuala Lumpur Malaysia; ^2^ Division of Tropical Health and Medicine James Cook University Townsville Australia; ^3^ School of Pharmacy International Medical University (IMU) Kuala Lumpur Malaysia; ^4^ School of Medicine International Medical University (IMU) Kuala Lumpur Malaysia; ^5^ University of Minnesota Minnesota

## 1 BACKGROUND

### 1.1 Description of the condition

The teaching and learning activities of any undergraduate curriculum will have a specific set of learning outcomes that should be successfully achieved by the students. The balance between the workload of a student and the available time to achieve the learning outcomes plays a major role in achieving these learning outcomes, as well as a good student satisfaction score and excellent final grades for that particular module (Whillier & Lystad, 2013). In a traditional educational experience, a teacher stands in front of the classroom, delivers a lecture to a group of students, who sit in rows, quietly listening to the lecture and taking notes. At the end of the lecture, students are given homework or an assignment to be completed outside of the classroom environment. This characterises the principle of “*sage‐on‐the stage”*, and is synonymous with the present day term of *teacher‐centered learning.* This is also referred to as the transmittal model (King, 1993), which assumes that the students are *passive note‐takers, receivers of the content or accumulators of factoids* (Morrison, 2014). Usually, the teacher does not have time to interact with the students individually during the class (Hamdan, McKnight, McKnight & Arfstorm, 2013), thus neglecting those students who do not understand the lecture. The traditional didactic way of teaching is primarily unidirectional and consists of limited interactions between the source of knowledge (teacher) and the passive recipients (students).

One of the main challenges faced by lecturers is the overload of academic content that needs to be taught in a relatively short period of time. Equally, the main challenge faced by the students is loss of interest or motivation to learn within the stipulated period of time (Prober & Khan, 2013). The traditional way of teaching, therefore, discourages the students from active learning and critical thinking. There is also increasing pressure from accreditation institutions, which demand “an ability to communicate effectively”, “an ability to identify, formulate and solve problems”, and “an ability to function on multidisciplinary teams” (Bishop & Verleger, 2013). As such, there is a need to transform the current pedagogical strategies, in order to enhance active learning in a more effective way (Al Faris et al., 2013). Synthesis of research on the effectiveness of lectures shows that lectures are not very effective for teaching and developing values or personal development, and may only be effective for the sole goal of transmitting information (Bligh, 2000). Taking these points together, it is important to explore methods that have the potential to maximise the use of classroom time and transform the classroom into a platform for teacher‐student interactions and critical thinking (Rui et al., 2017).

Numerous factors have cumulatively led to several challenges for traditional teaching in health professional education (HPE), including the availability of digital technologies, digitally‐empowered learners, the prolific expansion of courses, the amount of factual knowledge that has accumulated in the courses, prolific growth of health knowledge, advancement in healthcare disciplines, and investment into the scholarship of teaching and learning. To this end, newer delivery systems encompassing active learning in HPE have been developed. Studies have reported that active participation is an effective method to improve learning and understanding (Freeman et al., 2014; McCoy et al., 2015). Thus, to enhance interaction during their learning, there are educational strategies, which promote active learning in traditional lectures by engaging students in doing things and encouraging them to think about what they are doing. A classic example of active learning is a think–pair–share discussion, in which a student thinks individually for a moment about a question posed on the lecture, then pairs up with a classmate to discuss their ideas, and subsequently shares their answer with the entire class (King, 1993).

There are various modifications which can be incorporated into traditional lectures that enable active learning in the classroom, for instance; (a) the feedback lecture, which consists of two mini lectures separated by a small‐group study session built around a study guide, and (b) the guided lecture, in which students listen to a 20‐ to 30‐min presentation without taking notes, followed by their writing for 5 min on what they remember, and spending the remainder of the class duration in small groups for clarification and elaboration on the study material (Ellis, 2010; Johnson, 2013). Moreover, there are other active learning pedagogies, which include visual‐based instruction (Johnson et al., 2016), small group problem based learning, cooperative learning, debates, drama, role playing and simulation and peer teaching.

One innovative approach in education delivery system is the “flipped classroom,” an educational technique that consists of two parts, interactive group learning activities inside the classroom and direct personal computer‐based individual instruction outside the classroom (Bishop & Verleger, 2013). As such, work typically done as homework in the didactic model (e.g., problem solving, essay writing) is better undertaken in class with the guidance of the teacher. Listening to a lecture or watching videos is undertaken at home. Hence, the term *flipped* or *inverted classroom* is used (Herreid & Schiller, 2013). The essence of a flipped classroom is that the activities carried out during traditional class time and self‐study time are reversed or “flipped” (Veeramani, Madhugiri & Chand, 2015).

Approaches to undergraduate teaching have improved over the years as the scholarship of learning and teaching has provided evidence of what works to improve the outcomes. However, educational delivery approaches have shown little change in many disciplines and have remained the same for the majority of the sectors (Van Vliet, Winnips & Brouwer, 2015).

### 1.2 Description of the intervention

The flipped class is flexible itself and can be tailored (Tetreault, 2013). Historically, the concept of flipped classroom started in early 1990s. General Sylvanus Thayer created a system at West Point in USA, where a set of learning materials was given to engineering students so that they obtained core content prior to attending class. The classroom space was then used for critical thinking and group problem solving (Musallam, 2011). Many credited the rejuvenation of this idea with the development of, and increased access to, educational technologies (Moffett, 2015). For instance, the School of Business at the University of Miami proposed an ‘inverted classroom,’ which had events that traditionally took place inside the classroom now taking place outside the classroom and vice versa (Lage, Platt & Treglia, 2000). In 2000, a conference paper entitled ‘The Classroom Flip’ was presented by J Wesley Baker and the phrase ‘flipping the classroom’ was coined. Baker described how flipping the classroom could allow the trainer to become the ‘*guide on the side*’ rather than the ‘*sage on the stage*’ (Baker, 2000).

**Figure 1:**
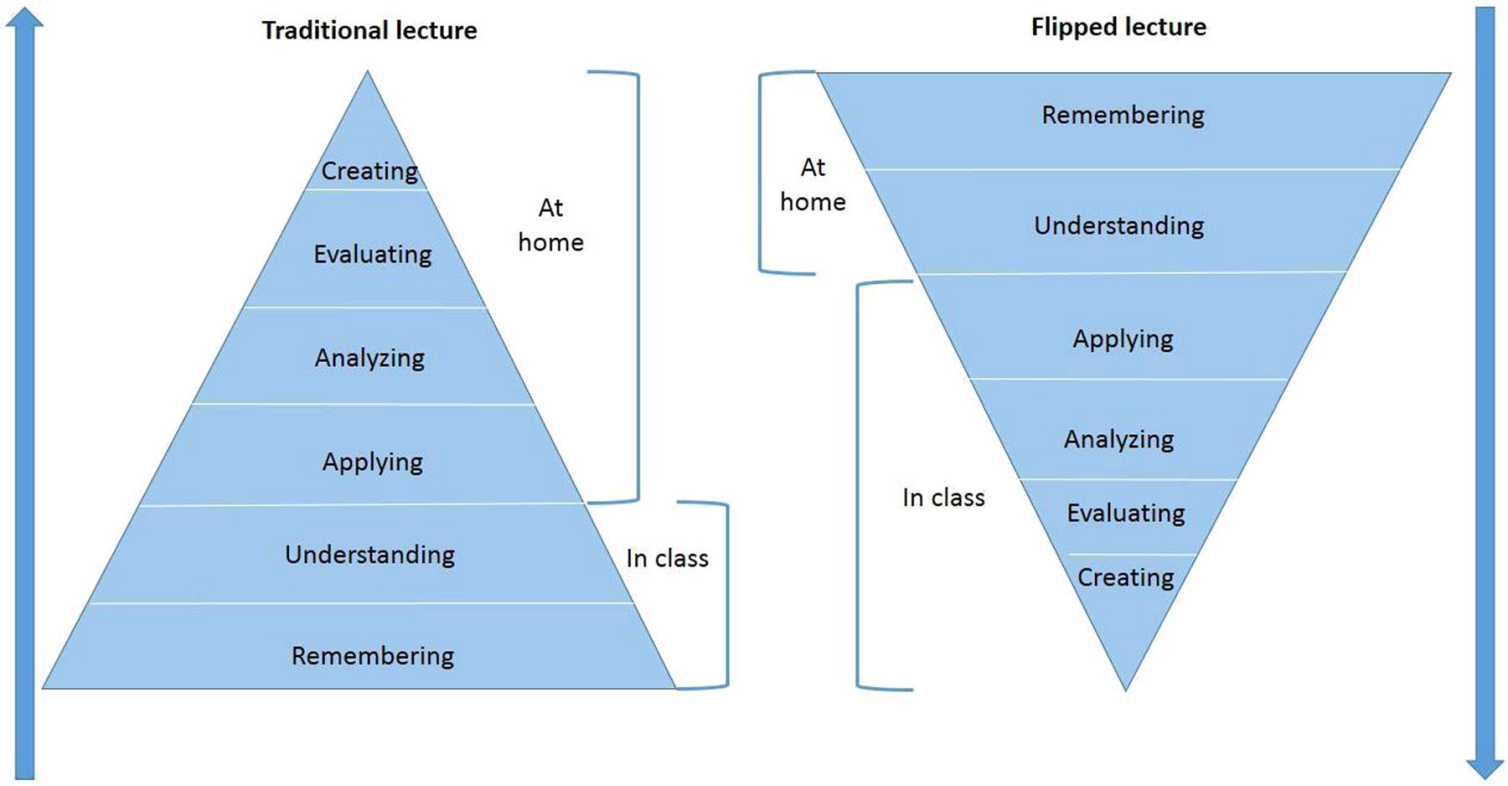
A comparison between the traditional learning and the flipped classroom in the Bloom's taxonomy [Color figure can be viewed at wileyonlinelibrary.com]

In a sense, this reversal also flips the Bloom's revised taxonomy because the lower level of cognitive work/knowledge acquisition is done by the students, while educators work interactively with the students to develop the higher forms of cognition (Figure 1). To date, this approach has attracted a large amount of attention in the HPE and a subsequent surge of literature.

Fundamentally, a flipped classroom encompasses two established elements of education, the recorded lecture (off campus learning) and active learning (on campus learning). Lectures are given as homework, as an aid to learning. Homework is important because it is a time where students can share their learning progress with their family, reflect on their learning, and review the material as well as the educator's feedback (Fulton, 2012). The key characteristics of a flipped classroom compared to a traditional classroom and other existing teaching methods are summarised in Table 1.

**Table 1:** Synopsis of the comparison between flipped classroom and other teaching modes

Description	Traditional classroom	Distant education	Flipped classroom
Teacher centred	√	√	‐
Student centred	‐	‐	√
Passive learning environment	√	√	‐
Active learning environment	√	√	√
Face‐to‐face lecture	√	‐	‐
First phase (Lecture)	In the classroom	At home	At home
Second phase (Active activities^a^)	At home	At home	In the classroom

^a^ Examples are group discussions, case studies, feedback sessions, problem solving activities, presentations and polling.

It has been highlighted that the flipped classroom fits into the broader context of blended learning (Tetreault, 2013). Blended learning as defined by Staker is ‘*a formal education program in which a student learns at least in part through online delivery of content and instruction with some element of student control over time, place, path, and/or pace and at least in part at a supervised brick‐and‐mortar location away from home*’(Staker & Horn, 2012, p.3). The flipped classroom consists of a formal education program, and online learning as a mechanism of informal learning through educational video quizzes/games. The flipped classroom approach is connected between what the students learn online (e.g., video lecture) and what they learn face‐to‐face (e.g., in‐class active case study), and vice versa, which is a common feature of blended learning (Tetreault, 2013). In principle, the flipped classroom assigns relatively low‐level cognitive learning such as memorising and understanding, outside of the classroom and teaching in class is accomplished mostly through teacher‐student interactions and cooperation between peers, thereby stimulating the students’ intellectual potential (Rui et al., 2017). The option to view video lectures (as an example) outside of classroom has beneficial effects for the learners as they can replay the videos as many times as needed to better understand the key concepts at their own pace. Furthermore, this allows each student to be able to comprehend the topics being covered to his/her satisfaction, whereas this might not be possible in the context of conventional teacher‐centred teaching. This is an important pedagogical consideration for international students for whom English is their second language (Moraros, Islam, Yu, Banow & Schindelka, 2015). From the teacher's perspective, a flipped classroom setting makes it easier to engage students and empower them as active participants of their own learning.

### 1.3 How the intervention might work

There are several theoretical constructs that are applicable for a flipped classroom. Two of these include: the technology acceptance model (TAM) (Davis, 1989) and the unified theory of acceptance and use of technology (UTAUT) (Venkatesh, Morris, Davis & Davis, 2003). These theoretical constructs provide a framework for the analysis and identification of relevant outcomes. We will outline how these two theories of flipped classroom learning can improve the learning outcomes such as student satisfaction and improved scores.

TAM includes two theoretical constructs: (a) perceived usefulness and (b) perceived ease of use. These constructs are defined as "the degree to which a person believes that using a particular system would enhance his or her job performance" and "the degree to which a person believes that using a particular system would be free of effort", respectively (Davis, 1989, p320). The first theoretical construct relies on students’ prior knowledge, gained from the pre‐class video lecture (for example), in enhancing their understanding (and overall learning performance) in the active in‐class activities such as problem solving. The second theoretical construct is based on students' perceptions that if a flipped class room is more user friendly than the traditional teaching mode, then they would be more likely to accept it.

The goal of the UTAUT model is to explain the intentions of a user to use a given information system and the subsequent behaviour of the user. The model is based on four primary constructs: 1) performance expectancy, 2) effort expectancy, 3) social influence, and 4) facilitating conditions (Venkatesh et al., 2003, p447). The first three constructs reflect the motivation of the users (i.e., students). The fourth construct reflects the characteristics of a flipped classroom setup when students engage with the pre‐class materials that are uploaded on an e‐learning portal. These material could be a video, an interactive presentation, a questionnaire or sometimes a recorded audio. With regard to these theoretical constructs, if students perceive that a flipped class room is user friendly and the academic environment facilitates their learning, then it will promote students' engagement, interactions and cooperation in learning, which will further improve their performance.

**Figure 2:**
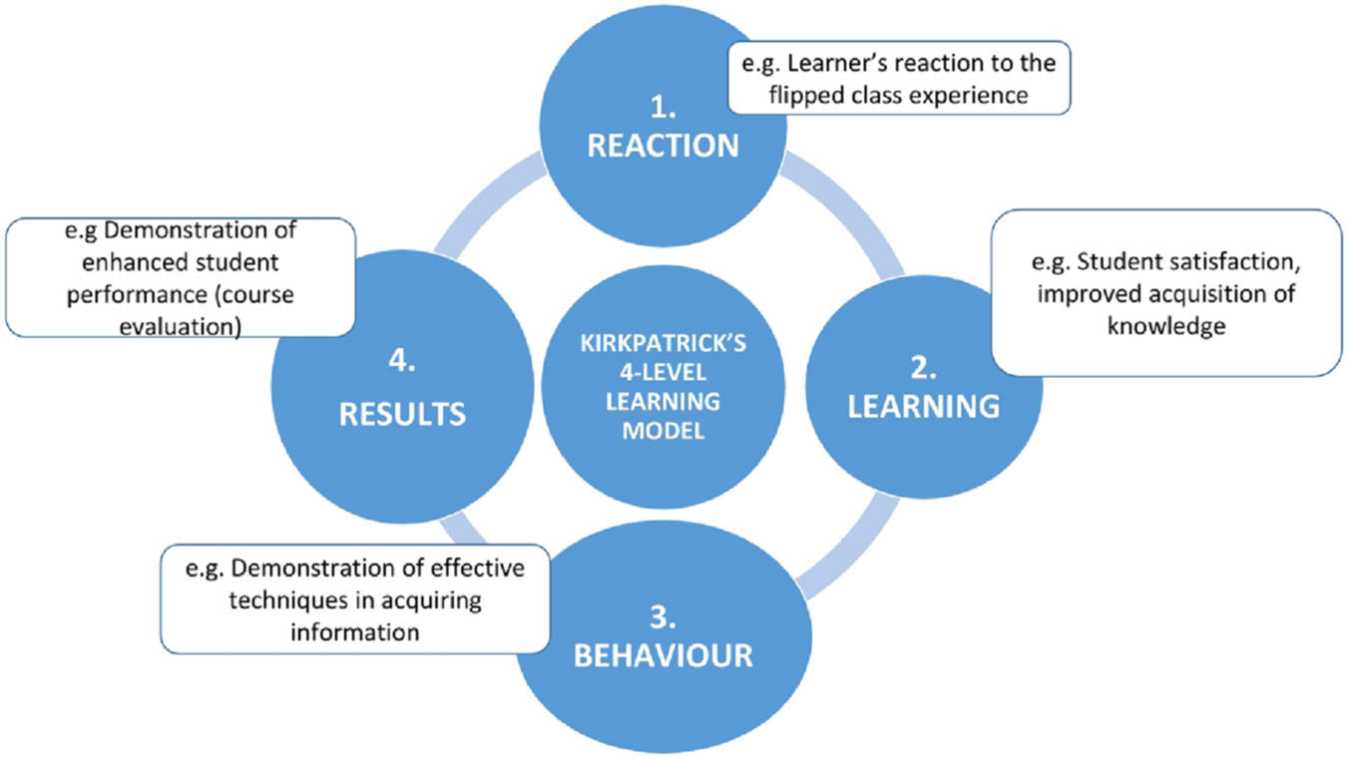
Four levels of learning in Kirkpatrick's model [Color figure can be viewed at wileyonlinelibrary.com]

There are potential advantages of a flipped classroom, including increased opportunities to provide individualised education to learners (Johnson, 2013; Kachka, 2012), increased student engagement with course material (Gross, Pietri, Anderson, Moyano‐Camihort & Graham, 2015), and increased educator‐student interaction, compared to a ‘performing’ lecture. The Kirkpatrick model of educational outcomes (Barry Issenberg, McGaghie, Petrusa, Lee Gordon & Scalese, 2005; Kirkpatrick & Kirkpatrick, 1994) comprises ‘learners’ reaction’ (to the educational experience); learning (modification of attitudes/perceptions and the acquisition of knowledge and skills); behaviour (self‐reported changes in practice and observed changes in practice, including new leadership positions); and results (which refers to change at the level of the organisation) (Figure 2). For instance, regarding the 'results' outcome, the flipped classroom allows the teacher to gain advanced, real‐time insight into how students learn and quickly identify and better address curriculum content that the students find most challenging. This insight can be used to better inform decisions with regard to effective curriculum organisation, structure and the delivery of future classes.

**Figure 3:**
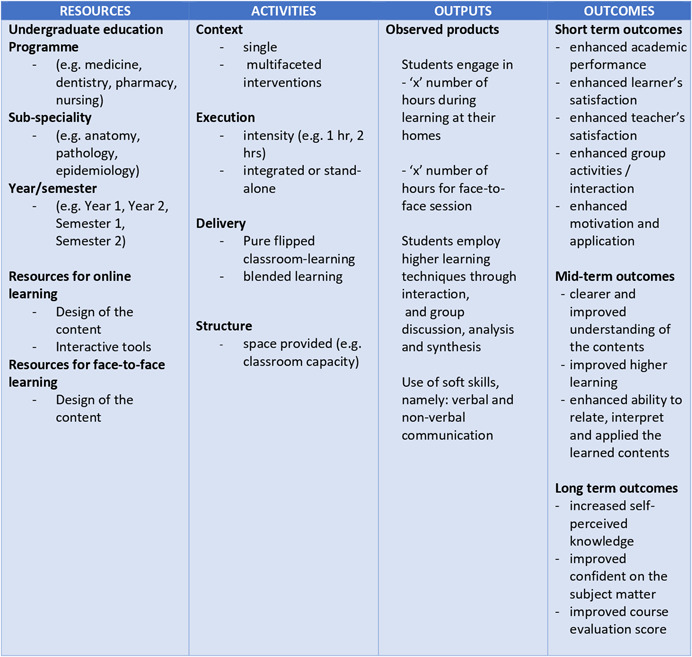
Logic model of flipped class learning [Color figure can be viewed at wileyonlinelibrary.com]

The success of a flipped classroom approach relies on a number of assumptions. Stimulation of students’ interest in learning and guided self‐study (Moraros et al., 2015), primarily depends on the opportunities to actively engage students in self‐directed learning and encourage progressive improvement (Bergmann, Overmyer & Wilie, 2012; Moraros et al., 2015) in assessment performances. Thus, a flipped class will not support effective learning if students fail to engage with the assigned pre‐class or in‐class activities (Kachka, 2012), for reasons which might include poorly designed educational materials (e.g., long, poor audio quality) or students feeling ‘lost’ (Moffett, 2015). As such, a number of contextual and structural factors that can influence flipped classroom learning include resources (inputs to the program), activities (aspects of implementation), outputs (observable products of the completed activities) and outcomes (effects or impacts within various time frames) as depicted in the conceptual framework (Figure 3).

### 1.4 Why it is important to do this review

There are individual studies, which have evaluated flipped classroom in medical education, allied health education and health science education, using a pre‐and post‐test design or comparative designs to explore how learning outcomes are improved. Some studies showed positive outcomes with flipped classroom (Galway, Corbett, Takaro, Tairyan & Frank, 2014; Van Vliet et al., 2015), while others showed the opposite (Whillier & Lystad, 2015). For instance, a study on integrated flipped lectures with online teaching techniques assessed learning experiences and participation through active learning. The findings suggested that the students in the integrated flipped‐online lectures had achieved an increase in active learning components compared to the group that were put in a didactic model (Galway et al., 2014). It is important to understand the factors that could have contributed to this difference. As an example, for balance of the safe learning environment (to be free from discomfort and fear) between the two groups of students, a comparability of the personality traits between the students in each group needs to be considered. On the other hand, another individual study, which assessed the effectiveness of flipped classroom in ophthalmology clerkship reported that the students in flipped classroom had more burden and pressure in preparing for the pre‐class compared with the students in lecturer‐based classroom group. Thus far, these published individual studies varied in design, sample size and outcome measures. It is unclear, if these findings would be generalised to other HPE. A non‐Campbell systematic review of the flipped classroom reported how the flipped classroom has been applied in nursing education and the achieved outcomes associated with such teaching (Betihavas, Bridgman, Kornhaber & Cross, 2016). Due to the focus on a particular educational context (i.e., nursing or ophthalmology), the generalisability of their findings to other courses in undergraduate HPE is uncertain. Another non‐Campbell collaborative systematic review, consisting of 82 studies reported on the effectiveness of flipped classroom in medical education where a pooled estimate of a subset of six experimental studies showed generally positive perceptions of the students to the flipped classroom. However, there were no significant changes in knowledge and skills (Cohen's *d* = −0.27 to 1.21, median: 0.08; Chen, Lui, & Martinelli, 2017). These systematic reviews, focused on a particular area (either nursing education or medical education) had a limited number of included studies, considerable variation in study designs, a lack of methodological quality assessment of the included studies, and the quality of evidence reported by these systematic reviews is poor.

A systematic review which combines the results of interventions, using flipped classroom compared with alternative learning or traditional learning, will help us to make recommendations for the development and implementation of successful flipped classroom amongst health professionals. The current review also aims to serve as a reference for decision makers to support evidence‐based approaches to flipped classroom in HPE.

## 2 OBJECTIVES

The primary objective of this systematic review is to assess the effectiveness of flipped classroom intervention for undergraduate health professional students on academic performance and course satisfaction.

The secondary objectives are to explore:

- The influence of context in the design, delivery and outcomes of the flipped classroom interventions in undergraduate health professional education;
- The barriers and facilitators of flipped classroom learning effectiveness for undergraduate health professional students.

Specifically, this review is designed to answer the following research questions:

### 2.1 Primary research question

What are the effects of flipped classroom learning on undergraduate health professional students' academic performance?

### 2.2 Secondary research questions

What are the effects of flipped classroom learning on undergraduate health professional students' course satisfaction?

Do any moderator variables affect the effectiveness of flipped classroom learning on academic performance outcomes?

Moderators will include (if data are available), study design, student related factors such as the amount of out‐of‐class preparation time, classroom availability and limited high speed internet access for rural and remote students, quality of interactive tools, and faculty related factors such as faculty members' preference to a more didactic approach.

## 3 METHODS

### 3.1 Criteria for considering studies for this review

#### 3.1.1 Types of studies

The following study designs are included in the review, as described in the Effective Practice and Organisation of Care (EPOC) group of the Cochrane Collaboration (EPOC 2017).

- Randomised designs, which include individual‐level randomised trials, cluster‐level randomised trials and natural experiments, where assignment to treatment or control conditions is functionally random.
- Non‐randomised designs, which include at least one treatment group and at least one comparison group, matching designs, two‐group pre‐post designs, regression discontinuity designs.

We do not include qualitative research.

#### 3.1.2 Types of participants

We included all undergraduate health professional students, regardless of the type of healthcare streams (e.g., medicine, dentistry, nursing, pharmacy), duration of the learning activity (e.g., one or two semesters) or the country where the study is conducted.

#### 3.1.3 Types of interventions

Any educational intervention that includes the flipped classroom as a teaching and learning activity in undergraduate programmes, regardless of the type of healthcare streams (e.g., medicine, dentistry, nursing, pharmacy) will be considered. To be included, a study must explicitly indicate that the teaching/learning activities for undergraduate students included in the flipped classroom, reversed classroom or flipping class, aiming to improve student learning and/or student satisfaction.

Standard lectures and subsequent tutorial formats will not be considered as flipped classroom. Studies on flipped classroom methods among undergraduate or postgraduate students who are not from the healthcare streams (e.g., engineering, economics, computer science) will be excluded.

#### 3.1.4 Types of outcome measures

We explored the impact of flipped classroom learning in undergraduate health professional students on academic related outcomes.

#### 3.1.5 Primary outcomes

The primary outcome is academic performance measured by examination scores, final grades or other formal assessment methods at immediate post‐test.

#### 3.1.6 Secondary outcomes

The secondary outcome is student satisfaction measured at immediate post‐test using a self report scale, which may include the training institution's own format of assessing student satisfaction.

### 3.2 Search methods for identification of studies

#### 3.2.1 Electronic searches

Following the guidelines of the Campbell Collaboration (Kugley et al., 2016), in order to retrieve a broad base of studies to review, an experienced 'Information Specialist' in this research team will search across an array of bibliographic databases, both in the subject area and in related disciplines.

A comprehensive and diverse search strategy will be used to search the relevant studies in the following databases.

1) Electronic databases
  a) MEDLINE,
  b) EMBASE,
  c) Education Resources Information Centre (ERIC),
  d) CINAHL,
  e) CENTRAL,
  f) SCOPUS,
  g) Best Evidence Medical Education,
  h) Web of Knowledge,
  i) Google Scholar,
  j) PsycInfo
  k) ProQuest (dissertation databases)
2) Research Registers and Websites
  a) Cochrane Collaboration Library
  b) Database of Abstracts of Reviews of Effectiveness
  c) System for Information on Grey Literature
  d) Evidence for Policy Practice Information and Coordinating Centre (EPPI‐Centre)
  e) Applied Social Sciences Index and Abstracts (ASSIA)
3) Dissertations and theses databases
  – Index to Theses in Great Britain and Ireland (www.theses.com/)
  – Theses Canada (www.collectionscanada.gc.ca/thesescanada/)
  – Networked Digital Library of Theses and Dissertations (http://www.ndltd.org/)
4) Regional bibliographic databases
  – Australia Australian Education Index (www.acer.edu.au/library/aei/index.html)
  – Britain British Education Index(www.leeds.ac.uk/bei/index.html)
  – Canada CBCA Education (www.twu.ca/Library/cbcaeduc.htm) Canadian Research Index (http://www.proquest.com/products‐services/canadian_research.html) ‐Latin America and the CaribbeanLILACS (health sciences) (http://lilacs.bvsalud.org/en/)
5) Full‐text journals available electronically
  – BioMedCentral (www.biomedcentral.com/browse/journals/)
  – Public Library of Science (PLoS) (www.plos.org/journals/)
  – PubMedCentral (PMC) (www.pubmedcentral.nih.gov/)
  – Directory of Open Access Journals (DOAJ) (www.doaj.org)
  – Education Research Global Observatory (http://ergo.asu.edu/ejdirectory.html)

### 3.3 Search terms

The following is an example of the types of terms we anticipate to use: 'undergraduate', 'flipped classroom', 'inverted classroom' 'health professional education'. In the final review, all searches actually used will be included so that they can be replicated. All search terms will be truncated using the convention appropriate for the given database so that they will include variations in endings of words and in spellings. Terms from the categories will be connected with “OR” within each category and by “ AND” between categories. We will consult the information specialist. Addressing studies from 2000 onward seems to strike a reasonable balance of covering various approaches to flipped classroom learning while primarily focusing on those that retain relevance in most recent educational practices. Ovid MEDLINE (R), ERIC via Ebsco and Education Source search strategy are provided in Appendix 1.

#### 3.3.1 Searching other resources

1) Grey literature sources
  a) Social Science Research Network
    We will look for the studies from the year 2000 onwards, regardless of language or study setting.
  b) Conference abstracts and proceedings such as American Educational Research Association Repository (http://www.aera.net/EventsMeetings/tabid/10063/Default.aspx) for 2013‐ 2017 will be reviewed to identify any potentially relevant studies.
2) Institutional repositories
  – Canadian Institutional Repositories http://www.carl‐abrc.ca/ir.html
  – Directory of Open Access Repositories (OpenDOAR)
  – Register of Open Access Repositories (ROAR)
3) Existing review and publication reference lists
  Reviews may also provide useful information about the search strategies used in their development. Copies of previously published reviews relevant to the current study will be obtained and checked for references to the included (and excluded) studies.
4) Ongoing studies
  We will also search the Social Care Online (http://www.scie‐socialcareonline.org.uk)
  We will contact the key researchers in the topic whether they have any studies in progress or unpublished research.
  Database searching will be supplemented by searches of the Web using Google (www.google.com) and Bing (www.bing.com) to locate additional articles.

#### 3.3.2 Manual search

We will also conduct a hand search of journals relevant to the topic. Example of such journals include

- American Educational Research Journal
- Journal of Educational Research

We will also review the reference lists of the relevant studies for any additional studies that have not been captured in the electronic databases.

### 3.4 Data collection and analysis

#### 3.4.1 Selection of studies

Two review authors (WSF and DKC) will independently screen the titles and abstracts identified according to the selection criteria for this review. Full‐text copies of all articles that might satisfy the inclusion criteria will be retrieved and reviewed for eligibility by WSF and DKC. Any disagreement will be resolved by consensus, and by referral to a third review author, if needed.

We will correspond with investigators of the primary studies, where necessary, to clarify study eligibility.

A PRISMA flow diagram (Moher, Liberati, Tetzlaff & Altman, 2009) will be used to summarise the study selection process and a table with the summary characteristics of excluded studies will be presented.

#### 3.4.2 Data extraction and management

For all studies eligible for this review, after the aforementioned screening process, two reviewers (DKC and WSF) will independently code all eligible studies using a piloted data extraction form. We will extract the following information from the included studies where possible.

1) Type of study design;
2) Study country;
3) Study setting (e.g. college/university/ institute, discipline);
4) Type of study participants (e.g. gender, age group, year at school);
5) Description of the education programme (e.g. duration of the flipped classroom, comparators, modality of intervention such as video lecture, YouTube lecture etc.);
6) Description of the comparator/any other interventions in addition to the education method;
7) Main outcomes (primary and secondary outcomes);
8) Outcome measurements (e.g. definition of outcome, tools used to measure outcome, time points of outcome measurement);
9) Any additional information that potentially affects the results.

Eligible studies will be coded on variables related to the above mentioned information that include study methods, the nature of the intervention and how it is implemented, the characteristics of the subject samples, the outcome variables and statistical findings, and contextual features such as country, setting, year of publication and so on. The coding manual providing the detailed instructions for coders in order to ensure consistency in selection of studies is provided in Appendix 2.

#### 3.4.3 Assessment of risk of bias in included studies

Risk of bias in the individual randomised trials will be analysed at the study level by using the Cochrane Risk of Bias tool (Higgins, Altman & Sterne, 2011). For non‐randomised designs, we will use the 'Risk of Bias' tool from the Cochrane Effective Practice and Organisation of Care Group (EPOC, 2009), which covers allocation sequence, similarity of baseline outcome measurement, similarity of baseline characteristics, incomplete outcome data, blinding of allocation, protection against contamination, selective outcome reporting and other risks of bias.

For most of the items, we will answer the following questions with ‘yes’ (low risk of bias), ‘no’ (high risk of bias) or ‘unclear’ (unclear risk of bias) to make judgments of risk of bias.

Data extraction (including methodological quality assessment) will be conducted independently by two reviewers (DKC and WSF). If there is any discrepancy, it will be resolved by taking a consensus between the two investigators. Otherwise, a third member of the review team (MAW) will be consulted to resolve the discrepancy.

We will present an overall grading of the evidence related to each of the main outcomes using the GRADE (Grades of Recommendation, Assessment, Development and Evaluation) approach. The GRADE approach defines the quality of a body of evidence as the extent to which one can be confident that an estimate of effect or association is close to the true quantity of a specific interest. The quality of a body of evidences involves the consideration of within trial risk of bias (methodological quality), directness of evidence, heterogeneity, precision of effect estimates, and risk of publication bias (Schünemann et al., 2011). A level of evidence for the “body of evidence” will be assigned, ranging from high, moderate, low to very low, as part of the GRADE process (Atkins et al., 2004). We will not exclude studies on the grounds of risk of bias, but sources of bias are reported when presenting the results of studies. We plan to present all included studies and provide a narrative discussion of risk of bias together with the potential limitations of the review as well as implications of bias in the interpretation of the results under the 'Discussion' section of the full text review.

#### 3.4.4 Unit of analysis issues

In cluster‐randomised trials, the unit of allocation is a group, rather than an individual. Since individuals within clusters tend to behave in a similar way, the data cannot be seen as being independent and thus have to be adjusted. A unit of analysis error typically arises if the study conducts analysis and programme placement at different levels and the analysis does not adequately account for this clustering (e.g., use cluster robust standard errors, variance components analysis). In such cases, the analysis would yield narrower confidence intervals than the true confidence intervals, increasing the risk of Type‐I error. This can be a problem in cluster randomised trials or in quasi‐experimental studies in which treatment allocation is clustered. For instance, participants within any one cluster (such as a semester) are often more likely to respond in a similar manner, and thus can no longer be assumed to act independently. This contributes to intra‐ cluster dependence (i.e., the intra‐class correlation).

In the event, when studies use cluster level assignment, we will adjust the standard errors of all effect size estimates using the method described in the Cochrane Handbook (Higgins, Deeks & Altman, 2011 ). If the intra‐class correlation needed to make this adjustment is not reported in the primary studies, we will use similar intra class correlations reported in other education trials (Hedges & Hedberg, 2007) and conduct sensitivity analyses using a range of plausible values. We will then include the data in the meta‐analysis.

If the cluster‐ randomised trials that we include sufficiently account for the cluster design, we will include the effect estimates in the meta‐analysis.

#### 3.4.5 Methods for handling dependent effect sizes

If the independence assumption is violated when studies produce several estimates based on the same individuals or there are clusters of studies that are not independent (such as those carried out by the same facilitator), we will use the robust variance estimator of the covariance matrix of meta‐regression coefficients, as described elsewhere (Hedges, Tipton & Johnson, 2010).

#### 3.4.6 Dealing with missing data

If there are missing standard deviations (SDs) for continuous outcomes, we will contact the corresponding author to see if data are available. If not available, we will calculate these using case‐analysis such as imputing SDs from standard errors (SEs), CIs, *t‐*values or *p* values (as appropriate) that are related to the differences between means in two groups, following the guidance described in the *Cochrane Handbook for Systematic Reviews of Interventions* (Higgins & Deeks et al., 2011).

When there is insufficient information available to calculate the SDs, we will impute SDs. If SDs available from other studies are included in this review for the change from baseline for the same outcome measures, we will use these as the missing SDs. If this approach is not applicable, assuming correlation coefficients from the two intervention groups are similar (this is reasonable for an randomised trial), we will impute SD of the change from baseline for the experimental intervention, following a formula described in the *Cochrane Handbook for Systematic Reviews of Interventions* (Higgins & Deeks et al., 2011).

If information is missing, for instance, SD, sample sizes or average outcomes in the comparison group follow‐up data collection, the missing data will be imputed from available information based on specific assumptions. The effect of missing data on the overall results will be assessed through sensitivity analysis by doing a meta‐analysis without imputing missing information.

#### 3.4.7 Assessment of heterogeneity

For the analysis of dichotomous and continuous data, an assessment of heterogeneity will be conducted. We will assess statistical heterogeneity using the *χ*2 test and the *I* ^*2*^ measure. The *χ*2 test assesses whether the observed differences in results are compatible with chance alone. The *I* ^*2*^ measure examines the percentage of total variation across studies due to (statistical) heterogeneity rather than to chance (Deeks, Higgins & Altman,  2011). The *I* ^2^ values will be used as a measure of presence of heterogeneity, which will be further explored (e.g., by using moderator analysis). Value of *I* ^*2*^ over 50% indicates the presence of a higher level of heterogeneity. In the absence of clinical heterogeneity and in the presence of statistical heterogeneity (where *I* ^*2*^ > 50%), we will choose a random‐effects model. Fixed effect meta‐analysis will not be applied because its homogeneity assumption is unlikely to be satisfied in this systematic review.

#### 3.4.8 Data synthesis

For dichotomous outcomes, risk ratio (RR) and its 95% confidence interval (CI) will be presented. This outcome, for example, includes students’ satisfaction (satisfied/not satisfied). We will conduct meta‐analyses, based on RRs and will summarise the results as a summary RR and its 95% CI.

For continuous outcomes such as mean and SD, we will use the mean difference (MD) or standardised mean difference (SMD) and their corresponding 95% CIs. SMD will be used if studies use different scale of measurement.

A SMD greater than zero or RR greater than 1 will indicate an increase in the outcome in the intervention group (flipped classroom) as compared to the comparison group.

In performing the meta‐analysis, we will synthesise the effect sizes for each outcome using the inverse‐variance random‐effects meta‐analysis.

Outcomes not measured numerically will be reported in a qualitative manner (e.g., factors affecting academic performance outcomes in flipped classroom).

We will either use RevMan (Review Manager, 2014), Stata's *metan* (Harris et al., 2008) and *metareg* commands (Harbord & Higgins, 2008), or the *metafor* package in R software (R Development Core Team, 2008; Viechtbauer 2010) to conduct the meta‐analysis, as appropriate. We will not combine evidence from different designs and outcome types in the same forest plot.

#### 3.4.9 Subgroup analysis and investigation of heterogeneity

Where possible, and if relevant, we will perform subgroup analyses to explore the influence of risk of bias on effect size. We will assess the influence of removing studies classed as having high and unclear risk of bias from meta‐analyses. These analyses will include only studies that are assessed as having low risk of bias.

If we identify a sufficient number of studies reporting the relevant data, we will conduct moderator analysis to determine whether the intervention effect significantly varies across study‐level, participant‐level or implementation‐related characteristics, including:

- Study design: Do randomised and non‐randomised designs exhibit consistently different effect sizes and significance values?
- Programme pathway (e.g., medicine, nursing, pharmacy)
- Sub‐speciality (e.g., ophthalmology, pharmacology, epidemiology)
- Amount of out‐of‐class preparation time
- Classroom availability and limited high speed Internet access for rural and remote students
- Quality of interactive tools used
- Faculty members' preference for a more didactic approach.

#### 3.4.10 Sensitivity analysis

We will perform the following series of sensitivity analysis:

- Removing studies with high and unclear risk of bias from the meta‐analyses. Therefore, the analysis would include only studies with low risk of bias in all key domains. This will show whether risks of bias has impact on the effect estimates.
- Using different plausible values for intra class correlation estimation for studies with cluster assignment.
- Inclusion of imputed data values to explore its impact on the effect estimates.

#### 3.4.11 Assessment of publication bias

We will use funnel plots to display the information about possible publication bias if we find sufficient studies (Higgins & Deeks et al., 2011). However, asymmetric funnel plots are not necessarily caused by publication bias (and publication bias does not necessarily cause asymmetry in a funnel plot). If asymmetry is present, we will consider the possible reasons for this.

## CONTRIBUTIONS OF AUTHORS

**Content**: Professor Maxine Whittake (Dean), Professor Cho Naing and Dr. Wong Shew Fung involved in developing curriculum in the respective medical programmes including flipped classroom learning. Dr. Dinesh Kumar is a faculty member involved in developing curriculum in allied health programmes. He has implemented flipped classroom learning in the Pharmacy programme.

**Systematic review methods**: Professor Maxine Whittaker and Professor Cho Naing have conducted and published several meta‐analyses in peer‐reviewed journals, including Cochrane systematic reviews, and have conducted WHO commissioned reviews. Dr. Wong Shew Fung and Dr. Dinesh Kumar have completed training for systematic reviews in the Cochrane context and are conducting a Cochrane systematic review.

**Statistical analysis**: Professor Maxine Whittaker and Professor Cho Naing have trainings and experience with teaching epidemiology and statistics that are applied in meta‐analyses.

**Information retrieval**: Librarian Amy Riegelman has training and experience with developing search methods for the purposes of evidence synthesis. In her role at the University of Minnesota, she is the subject liaison to the following departments: Educational Psychology, Psychology, Child Development, and Speech‐Language‐Hearing‐Sciences. Amy is on the Campbell Information Retrieval Group and co‐chairs a systematic review service at the University of Minnesota.

## DECLARATIONS OF INTEREST

None

## SOURCES OF SUPPORT

### Internal sources

- MAW and CN: College of Public Health, Medical and Veterinary Sciences, James Cook University, Townsville, Australia

### External sources

- No sources of support provided

## APPENDIX A

### Search strategy

**Ovid MEDLINE (R)**

1. exp Education, Dental/ OR exp Education, Medical, Undergraduate/ OR exp education, medical/ OR exp Education, Nursing, Baccalaureate/ OR exp education, nursing/ OR exp education, pharmacy/ OR exp education, predental/ OR exp education, premedical/ OR exp education, professional/ OR exp education, public health professional/

1. exp Health Occupations/
2. exp education/
3. AND 3
4. 1 OR 4
5. (anatomy OR BSN OR "health profession*" OR chiropract* OR dental OR Medical OR Nurs* OR pre#dental OR pre#med* OR pharmac* OR "public health").tw.
6. (bachelor* OR class* OR course* OR educat* OR learn* OR instruct* OR professor* OR student* OR teach* OR train* OR undergrad*).tw.
7. 6 AND 7
8. 5 OR 8
9. (("flip* the class*") OR (flipped#classroom) OR (flip* ADJ10 class*) OR (flip* ADJ10 educat*) OR (flip* ADJ10 learn*) OR (flip* ADJ10 instruct*) OR (flip* ADJ10 teach*)).tw
10. (("invert* the class*") OR (inverted#classroom) OR (invert* ADJ10 class*) OR (invert* ADJ10 educat*) OR (invert* ADJ10 learn*) OR (invert* ADJ10 instruct*) OR (invert* ADJ10 teach*)).tw
11. (invertebrate* or flippase*).tw.
12. 10 OR 11
13. 13 NOT 12
14. 9 AND 14

Total: 367

Timestamp: 20190127

**ERIC via Ebsco**

1. SU (“Allied Health Occupations Education” OR “Allied Health Education” OR “Health Occupations Education” OR “Nursing Students” OR “Medical Education” OR “Nursing Education” OR “Medical Education” OR “Premedical Students”)
2. TI (anatomy OR BSN OR chiropract* OR dental OR "health profession*" OR Medical OR Nurs* OR pharmac* OR pre#dental OR pre#med* OR "public health")
3. AB (anatomy OR BSN OR chiropract* OR dental OR "health profession*" OR Medical OR Nurs* OR pharmac* OR pre#dental OR pre#med* OR "public health")
4. OR 3
5. TI (bachelor* OR class* OR course* OR educat* OR learn* OR instruct* OR professor* OR student* OR teach* OR train* OR undergrad*)
6. AB (bachelor* OR class* OR course* OR educat* OR learn* OR instruct* OR professor* OR student* OR teach* OR train* OR undergrad*)
7. SU (“Higher Education” OR “College Faculty” OR “Undergraduate Study” OR “College Students” OR “College Instruction”)
8. 5 OR 6 OR 7
9. AND 8
10. 1 OR 9
11. TI (("flip* the class*") OR (flipped#classroom) OR (flip* N10 class*) OR (flip* N10 educat*) OR (flip* N10 learn*) OR (flip* N10 instruct*) OR (flip* N10 teach*))
12. AB (("flip* the class*") OR (flipped#classroom) OR (flip* N10 class*) OR (flip* N10 educat*) OR (flip* N10 learn*) OR (flip* N10 instruct*) OR (flip* N10 teach*))
13. TI (("invert* the class*") OR (inverted#classroom) OR (invert* N10 class*) OR (invert* N10 educat*) OR (invert* N10 learn*) OR (invert* N10 instruct*) OR (invert* N10 teach*))
14. AB (("invert* the class*") OR (inverted#classroom) OR (invert* N10 class*) OR (invert* N10 educat*) OR (invert* N10 learn*) OR (invert* N10 instruct*) OR (invert* N10 teach*))
15. 11 OR 12 OR 13 OR 14
16. 10 AND 15

Total: 42

Timestamp: 20190128

**Education Source**

1. SU (“Allied health personnel” OR “Medical personnel” OR “Premedical education” OR “Premedical students” OR “Chiropractic students" OR "Chiropractic schools" OR "Chiropractic education" OR "Chiropractic students" OR "Nursing Education" OR "Baccalaureate nursing education" OR "Public health education (Higher)" OR "Publish health nursing education" OR "Public health education" OR "Education of public health nurses")
2. TI (anatomy OR BSN OR chiropract* OR dental OR "health profession*" OR Medical OR Nurs* OR pharmac* OR pre#dental OR pre#med* OR "public health")
3. AB (anatomy OR BSN OR chiropract* OR dental OR "health profession*" OR Medical OR Nurs* OR pharmac* OR pre#dental OR pre#med* OR "public health")
4. OR 3
5. TI (bachelor* OR class* OR course* OR educat* OR learn* OR instruct* OR professor* OR student* OR teach* OR train* OR undergrad*)
6. AB (bachelor* OR class* OR course* OR educat* OR learn* OR instruct* OR professor* OR student* OR teach* OR train* OR undergrad*)
7. SU (Undergraduates OR "Undergraduate programs" OR "Bachelor's degree" OR "University faculty" OR "College teachers")
8. 5 OR 6 OR 7
9. AND 8
10. 1 OR 9
11. SU (Flipped classrooms)
12. TI (("flip* the class*") OR (flipped#classroom) OR (flip* N10 class*) OR (flip* N10 educat*) OR (flip* N10 learn*) OR (flip* N10 instruct*) OR (flip* N10 teach*))
13. AB (("flip* the class*") OR (flipped#classroom) OR (flip* N10 class*) OR (flip* N10 educat*) OR (flip* N10 learn*) OR (flip* N10 instruct*) OR (flip* N10 teach*))
14. TI (("invert* the class*") OR (inverted#classroom) OR (invert* N10 class*) OR (invert* N10 educat*) OR (invert* N10 learn*) OR (invert* N10 instruct*) OR (invert* N10 teach*))
15. AB (("invert* the class*") OR (inverted#classroom) OR (invert* N10 class*) OR (invert* N10 educat*) OR (invert* N10 learn*) OR (invert* N10 instruct*) OR (invert* N10 teach*))
16. 11 OR 12 OR 13 OR 14 OR 15
17. 10 AND 16

Total: 145

Timestamp: 20190128

## APPENDIX B

### Coding manual for study eligibility

**Table:**

Reference ID:			Reviewer ID:		
			Date:		
**Title of Article**:					
**Type of publication:**					
**Please, tick ƴ**					
(i) Journal article					
(ii) Book					
(iii) Book chapter (in an edited book)					
(iv) Thesis or dissertation					
(v) Technical report					
(vi) Conference paper / presentation					
(vii) Others: Specify: ‐‐‐‐‐‐‐‐‐‐‐‐‐‐‐‐‐‐‐‐‐‐‐‐					
**Author**:			**Year of Publication**:		
**Name of Publisher:**		**Volume:**		**Page No:**	
**Country in which the study was conducted:**					
		**Yes**	**No**	**Unclear**	**Remarks**
1.	**Study Design**				
	What Is the study? **Plese, tick ƴ**				
	1. Randomised trial				
	2. Cluster randomised trial				
	3. Non‐randomised trial (Plese, specify)				
	Were the participants randomly assigned into intervention or control group?				
	Was the allocation adequately concealed from each participant?				
2.	**Participants**				
	Did the study include adults pursuing undergraduate healthcare programme?				
	Did the study include flipped classroom in the delivery of the undergraduate healthcare curriculum?				
	Did the study include lecture‐based learning or conventional teacher‐centred learning in the delivery of the undergraduate healthcare curriculum?				
	Was the level of understanding of each participant on the contents be assessed prior to the intervention?				
	Was the level of understanding of each participant on the contents be assessed after the intervention?				
3.	**Interventions**				
	Did the intervention group receive same preparatory materials for the lecture as the control group?				
	Did the intervention and control groups receive similar support for learning (e.g. guidance from teacher)?				
	Did number of data points before and after intervention describe?				
	Plese, specify.....				
4.	**Outcomes**				
	Was the outcome evaluation blinded?				
	**• What were the measured outcomes? Please, tick ƴ**				
	1) Scores				
	2) Improved knowledge or skills				
	3) Increased motivation to learn				
	4) Increased time spent to prepare for the class				
	5) Increased engagement by the lecturer				
	6) Increased social interaction with other learners				
	7) More enjoyable in learning				
	8) Fewer distractions				
	9) The knowledge learnt is more applicable				
	10) Better retention of learned materials				
	11) Strengthen the learner analytical skills				
	12) Others: Specify: ‐‐‐‐‐‐‐‐‐‐‐‐‐‐‐‐‐‐‐‐‐‐‐‐				
	**• Was Kirkpatrick Framework used for the measure of the effectiveness of the outcomes? Please, tick ƴ**				
	1) Perceptions of intervention				
	2) Attitude changes				
	2) Changes of knowledge and skills				
	3) Changes in behaviours				
	4) Changes in professional practice				
	5) Changes in patient outcomes				
	6) Others Specify: ‐‐‐‐‐‐‐‐‐‐‐‐‐‐‐‐‐‐‐‐‐‐‐‐				
		**Yes**	**No**	**Unclear**	**Remarks**
5.	Have ethical issues been taken into consideration?				
		**Yes**	**No**	**Unclear**	**Remarks**
6.	Should this study be included in the review?				
